# Separation and Elevated Residential Mobility: A Cross-Country Comparison

**DOI:** 10.1007/s10680-020-09561-1

**Published:** 2020-05-29

**Authors:** Hill Kulu, Júlia Mikolai, Michael J. Thomas, Sergi Vidal, Christine Schnor, Didier Willaert, Fieke H. L. Visser, Clara H. Mulder

**Affiliations:** 1 University of St Andrews, St Andrews, UK; 2 Population Research Centre, Faculty of Spatial Sciences, University of Groningen, Groningen, The Netherlands; 3 Statistics Norway, Oslo, Norway; 4 Centre for Demographic Studies, Universitat Autònoma de Barcelona, Barcelona, Spain; 5 Université Catholique de Louvain, Louvain-la-Neuve, Belgium; 6 Vrije Universiteit Brussel, Brussel, Belgium; 7 Department of Economic Geography, Faculty of Spatial Sciences, University of Groningen, Groningen, The Netherlands

**Keywords:** Divorce, Separation, Residential mobility, Migration, Poisson regression, Cross-national comparison, Housing markets

## Abstract

This study investigates the magnitude and persistence of elevated post-separation residential mobility (i.e. residential instability) in five countries (Australia, Belgium, Germany, the Netherlands, and the UK) with similar levels of economic development, but different welfare provisions and housing markets. While many studies examine residential changes related to separation in selected individual countries, only very few have compared patterns across countries. Using longitudinal data and applying Poisson regression models, we study the risk of a move of separated men and women compared with cohabiting and married individuals. We use time since separation to distinguish between moves due to separation and moves of separated individuals. Our analysis shows that separated men and women are significantly more likely to move than cohabiting and married individuals. The risk of a residential change is the highest shortly after separation, and it decreases with duration since separation. However, the magnitude of this decline varies by country. In Belgium, mobility rates remain elevated for a long period after separation, whereas in the Netherlands, post-separation residential instability appears brief, with mobility rates declining rapidly. The results suggest that housing markets are likely to shape the residential mobility of separated individuals. In countries, where mortgages are easy to access and affordable rental properties are widespread, separated individuals can rapidly adjust their housing to new family circumstances; in contrast, in countries with limited access to homeownership and small social rental markets, separated individuals experience a prolonged period of residential instability.

## Introduction

Destandardisation, discontinuity, uncertainty, and instability are all terms that describe individual and family life courses within contemporary Western societies (e.g. Beck 1992; Giddens 1992; Huinink 2013; Lesthaeghe 2010; Lesthaeghe and van de Kaa 1986). The significant rise in separation and divorce rates is a clear reflection of such processes: breaking normative life-course trajectories, leading to diverse and destandardised post-separation households and often marking the beginning of a period of increased instability.

A large body of the literature has emerged to investigate the effect of separation on economic (e.g. Andreß et al. 2006; Uunk 2004) and material/subjective well-being (e.g. Kalmijn 2009; Osborne et al. 2012). As the breakdown of a co-residential partnership necessitates the spatial relocation of at least one ex-partner (and potentially both), a further area of research has developed with a focus on the residential mobility and housing consequences of partnership and family breakdown (e.g. Clark 2013; Cooke et al. 2016; Courgeau 1985; Gram-Hanssen and Bech-Danielsen 2008; Mulder and Malmberg 2011; Mulder et al. 2012; Mulder and Wagner 2010, 2012; Wasoff and Dobash 1990). Because moves related to separation are usually urgent and financially restricted (Feijten and van Ham 2007), separated individuals are more likely to move to smaller, lower-quality dwellings (Feijten 2005; Gober 1992) and over shorter distances than those in intact couple relationships (Feijten and van Ham 2007). The interdependencies inherent in the life course mean that persistent post-separation residential instability could lead to instability in other important life domains including those of ‘significant others’ (e.g. children).

This paper investigates the magnitude and persistence of elevated post-separation residential mobility (or residential instability). We extend previous research in the following ways. First, we conduct a comparative study of five high-income countries: Australia, Belgium, Germany, the Netherlands, and the UK. While previous research has investigated residential and housing changes related to separation in selected individual countries, only a few studies have compared trends and patterns in post-separation outcomes across countries (e.g. Dewilde 2008; Lersch and Vidal 2014; Mikolai et al. 2019). Wider structural differences in welfare state provisions and housing markets across countries are likely to be central in shaping and constraining individuals’ opportunities to access suitable housing and to ‘recover’ in terms of residential stability. Through a comparison of countries with similar levels of economic development, but different welfare state traditions, family policies, mortgage systems, and housing markets, we offer novel empirical insights into the potential role of such factors in post-separation residential (in)stability.

Second, we distinguish between moves due to separation and moves of separated individuals. Although previous studies have discussed the issue, they have only analysed moves of separated individuals. We use time since separation to distinguish the two types of moves, which, we believe, is a novel way of addressing this issue. By distinguishing between these two types of moves, and including them both in the analyses, we are able to study short- and long-term effects of separation on individuals’ residential trajectories. This will improve our understanding of the degree to which separated individuals experience relatively short or more prolonged phases of residential instability.

Finally, this study shows an innovative way of conducting a comparative study of spatial mobility of separated individuals when individual-level data cannot be shared across research teams because of confidentiality issues. We make use of the count-data approach (Hoem et al. 2010) for event history analysis and fit a Poisson regression model on cross-tabulated occurrence–exposure data. This approach is equivalent to estimating event history models on individual-level data; it can be used when only occurrence–exposure data aggregated over time intervals and covariate categories can be shared across countries.

## Separation and Elevated Residential Mobility

### Why do Separated People Move?

Residential mobility enables individuals to adjust their living conditions as their circumstances change across the life course (Wagner and Mulder 2015). Because moving is costly, individuals will normally only move if the benefits of a move outweigh its costs (Feijten and Mulder 2010). Moves can be triggered by a range of different events such as dissatisfaction with the current dwelling or neighbourhood (Coulter and Scott 2015), job opportunities (van Ham 2002), or family life events such as union formation, marriage, or childbirth. Moves related to family formation are associated with moves to larger, better quality dwellings, and often to homeownership as individuals adjust their housing circumstances to their needs and preferences associated with a larger family (e.g. Clark and Davies Withers 2009; Clark and Huang 2003; Davies Withers 1998; Feijten and Mulder 2002; Helderman et al. 2004; Kulu 2008; Kulu and Steele 2013; Michielin and Mulder 2008; Mulder and Lauster 2010). Residential mobility decisions are the outcome of a set of complex interrelationships between individuals’ desires, intentions (Clark and Lisowski 2017a, b; Coulter et al. 2011; de Groot et al. 2011; Kley 2011), resources, and structural constraints (Wagner and Mulder 2015).

Moves related to separation are driven by different reasons than moves related to family formation. First, by definition, separation requires at least one ex-partner to move out of the joint home. Such moves tend to occur within a context of temporal urgency and financial constraints (Feijten and van Ham 2007). Union dissolution implies that separated individuals become single-earner households, no longer benefit from economies of scale, and have to divide their assets (Feijten and Mulder 2005). Together, these conditions can contribute to an increased likelihood of moving into less desirable residential settings and housing conditions (Feijten 2005; Flowerdew and Al-Hamad 2004; Sullivan 1986). Additionally, many moves related to separation are non-voluntary (Mulder 1996), undesired, or unintended. Previous empirical research in the USA (Clark and Lisowski 2017b), Australia (Clark and Lisowski 2017a), and the Netherlands (de Groot et al. 2011) has shown that most moves among individuals who had not intended to move were triggered by separation. Additionally, union dissolution was shown to have a large positive effect on expecting an undesired move in the UK (Coulter et al. 2011). Unexpected mobility can lead to affordability problems and a disconnection from family and friends (Clark and Lisowski 2017a).

Second, the effect of separation on residential mobility might be long-lasting. It is possible that an ex-partner who initially remained in the joint home will eventually have to move out either because they cannot afford to continue to pay for the costs of housing and maintenance independently (Feijten 2005) or because the sale of the ex-couple’s joint home was a lengthy process (Feijten and Mulder 2005). In both cases, these moves are delayed initial moves out of the former joint home. Separation can also have a long-term effect on residential mobility if individuals who moved out of the joint home (either at the time of separation or later) experience several ‘adjustment’ moves as they search for an appropriate dwelling. If increased mobility levels persist among separated individuals, it may be indicative of a phase of undesirable post-separation residential instability with repeated adjustment moves undertaken within particularly constrained opportunity structures. The occurrence of prolonged residential instability could be expected to have deleterious consequences for a range of other life domains. For instance, residential instability could contribute to corresponding instabilities in individuals’ psychological well-being (Oishi 2010), the formation and maintenance of partnerships (Boyle et al. 2008), resident children’s schooling and access to friendship networks (South and Haynie 2004), and, more broadly, post-separation social and economic status.

Many separated individuals form a new partnership. However, moves related to repartnering are driven by different motives and circumstances than moves related to separation. These moves are similar to moves related to union formation (Mikolai and Kulu 2018a). Therefore, individuals who repartner following separation are treated as married or cohabiting in this study. Hence, the term ‘separated’ denotes individuals who separate and remain separated (i.e. do not repartner) during the observation period.

### Cross-National Differences in Separation and Elevated Residential Mobility

Much of the empirical literature on separation and residential mobility focuses on individual and household characteristics. The omission of wider structural influences could be particularly limiting in the context of post-separation residential mobility and instability (Findlay et al. 2015). Indeed, while rare, previous comparative work on separation and residential mobility suggests that variations in welfare state traditions, family policies, and housing markets may play a crucial role in mitigating the consequences of separation and divorce across countries (Dewilde 2008; Lersch and Vidal 2014; Mikolai et al. 2019; Thomas and Mulder 2016; Uunk 2004). This study examines variations in post-separation residential instability across five Western countries: Australia, Belgium, Germany, the Netherlands, and the UK. These countries are similar in their levels of economic development, but differ in their housing-market traditions, configurations, and policies.

Taking into consideration the differing approaches to the family, the market, and the state, Esping-Andersen’s (1990, 1999) welfare state typology has often formed the conceptual framework for cross-national comparisons. More recently, Mulder and Billari (2010) have proposed a typology of homeownership regimes, which, although aimed at explaining fertility variations across countries, may hold particular potential for comparative analyses of residential mobility and instability. In the post-separation context, the ability to make successful ‘adjustment’ moves and recover residential stability is likely to be strongly linked to the accessibility of suitable and affordable housing.

Mulder and Billari (2010) distinguish four types of homeownership regimes. In the ‘career homeownership’ regime, mortgages are widespread and represent a major source of financing homeownership. Yet, homeownership is not universal, nor is it always considered normative; rather it represents a progressive step in the housing career for those who have sufficient and stable incomes. As such, renting can be deemed an acceptable alternative, though mostly for singles and childless couples, while homeownership is traditionally reserved for families. A number of Western and Northern European countries (e.g. Denmark, Germany, the Netherlands, Sweden, and the UK) belong to this housing regime. By contrast, the ‘elite homeownership’ regime is characterised by constrained access to mortgages, and the requirement for housing costs to be financed from savings, family help, or inheritance. Consequently, homeownership tends to be restricted to more affluent families. In this context, renting is both an acceptable and necessary alternative. Austria, Belgium, and France provide examples of this group. The ‘easy homeownership’ regime is characterised by widely available mortgages and high levels of homeownership. This regime includes selected Northern European countries (e.g. Finland, Norway, and Iceland). Finally, the ‘difficult homeownership’ regime represents those countries where homeownership is normatively prescribed and extremely common, but access to mortgages is limited and the rental sector is poorly developed. Again, personal savings, family help, and inheritance largely determine access to homeownership. Southern European countries make up this group. Although Australia was not originally included in this typology, it resembles the characteristics of the ‘career homeownership’ regime (Beer et al. 2007; Yates 2008).

While access to homeownership is likely to be an important factor in regaining post-separation residential stability, it is important to consider the alternatives available for those who cannot afford, or do not wish, to (re-)enter homeownership. Kemeny (2001) distinguished two types of housing markets based on whether rental accommodation is publicly or privately owned. In ‘dualist’ rental markets (e.g. the UK, Belgium, and Australia), some of the rental stock is in private hands, while some of it is publicly owned. There is no competition between the public and private market in this housing market, because publicly owned, socially rented accommodation is available only to those in need. Private renting in these countries tends to be linked to lower socio-economic status and to a lower price/quality ratio (Dewilde 2017). In these countries, homeownership dominates and there are no attractive alternatives in the rental market (Lersch and Dewilde 2015). By contrast, in ‘unitary’ rental markets (e.g. Germany and the Netherlands), access to public housing is not or only partly restricted by need, and competition between the private and public sector is encouraged (Kemeny 2001). This competition has resulted in good-quality housing across all types of housing tenures and income groups (Dewilde 2017) and attractive alternatives to homeownership in the rental market (Lersch and Dewilde 2015).

Where the characteristics of the housing market do not facilitate relatively urgent moves into good-quality dwellings, it may be an option for separated individuals to move back to their parents’ place at least temporarily (Arundel and Lennartz 2017). Although the study does not include countries with strong family ties and intergenerational family support (e.g. Southern European countries), recent studies show that moving to the parents’ place is also common in other countries (e.g. Albertini et al. 2018; Das et al. 2017; Murinkó 2019; Stone et al. 2014). Particularly men, those with low income, and younger people are likely to move (back) to their parents’ home following separation. Overall, the share of extended-family households is low across the study countries (Iacovou and Skew 2011); in 2008, it ranged from 0.1% in the Netherlands to 1.3% in the UK.

A recent study by Dewilde (2017) has empirically tested the validity of the commonly used housing-market typologies. She performed cluster analysis on 15 European countries using data on various housing-market characteristics. The study showed that more state intervention to regulate the rental market was associated with better housing conditions and lower housing costs. This was especially the case in countries with a unitary rental market and a social-democratic welfare regime (e.g. the Netherlands). Countries with a unitary rental market but a conservative welfare regime (e.g. Germany) tended to cluster together with those countries that have a dual rental market (e.g. UK). She found no evidence that low- to moderate-income renters are better off in countries with a large social housing sector than in countries with a large private rental sector and they were found to be the worst off in countries where the size of the social renting sector is small (e.g. Belgium). This is because in these countries, fewer low-income individuals will have access to social housing.

The characteristics of the homeownership and rental market are likely to have different implications for separated individuals’ post-separation residential mobility because different contexts offer different opportunities for tenure stability, and for finding affordable, good-quality housing. The motives of separated individuals to move are expected to be similar across countries for the initial move. However, in housing markets where it is easier to find a suitable and affordable dwelling following separation, individuals will have to make fewer adjustment moves and will, thus, be likely to reach residential stability earlier than in countries where the rental market offers dwellings of sub-optimal quality and/or price.

Combining information from the aforementioned typologies, the Netherlands is expected to offer the best housing conditions at affordable prices to separated individuals who have to move out of the joint home upon separation on short notice. Because of the characteristics of the housing market, separated individuals in the Netherlands may not need to make (as many) ‘adjustment’ moves following separation. At the other end of the spectrum is Belgium with a small social rental sector, restricted access to homeownership, and limited opportunities for individuals with low- to middle-income. This implies that in Belgium, separated individuals may only be able to find lower-quality and/or expensive housing and it may take time and several ‘adjustment’ moves before individuals experience an improvement in their financial and housing circumstances. The UK and Australia are expected to hold an intermediate position, with less regulated private rental sectors, shorter contracts, less security of tenure, and a social renting sector which is larger but restricted to those in need. We also expect Germany to belong to this group, although it has a large, well-regulated rental sector with affordable prices for disadvantaged population subgroups.

Differences in housing markets across countries are not the only factor which is likely to influence separated individuals’ residential (in)stability. Cross-national differences in welfare states, family policies, and the level of gender equality are also likely to influence the relationship between separation and post-separation residential mobility. The studied countries are similar with respect to trends in partnership formation and dissolution. For example, in all five countries, cohabitation has replaced marriage as a first union (OECD 2016). Cohabiting unions are likely to end in marriage or in separation—long-term cohabitation is still rare; 10 years after the formation of a cohabiting union, around 37% dissolve in Germany, 35% in Belgium, 26% in the Netherlands (Andersson et al. 2017), and 30% in the UK (Ermisch and Francesconi 2000). In Australia, 40% of cohabitations formed between 1990 and 1994 dissolved in 5 years following separation (de Vaus 2004). Marital separation rates have also increased. After 10 years, 11% of marriages end in divorce in Germany and the Netherlands, 17% in Belgium (Andersson et al. 2017), 21% in the UK (Hannemann and Kulu 2015) and 26% in Australia (de Vaus 2004). These estimates are based on survey data and cover slightly different periods across countries.

The study countries are also relatively similar regarding their legislation on dividing assets and savings as well as the custody of children following divorce (see European e-Justice Portal^1^ for European countries and Family Law Act 1975 for Australia for more information). Overall, following divorce, couples are encouraged to agree between themselves on how to divide their assets and savings, and the responsibility to care for their children. If the couple does not come to an agreement, the courts get involved. The Netherlands represents an unusual case, where at the time of data collection by default, all property became jointly owned after marriage regardless of whether it was acquired before or after marriage. Following divorce, this joint property is divided equally unless the parties have agreed otherwise. If the couple has joint children, joint custody is encouraged following divorce in most countries. If the ex-spouses can agree on custody arrangements and maintenance fees, the courts do not get involved. If this is not the case, the courts get involved and make a decision, which prioritises the interest and rights of the child(ren). In countries where cohabitation can be registered and registered partnerships are considered as a legal category by law (the Netherlands and Belgium), the rights of married and registered cohabiting couples are the same. In the study, countries where cohabitation cannot be legally registered, the law does not regulate the rights and responsibilities of separating cohabiting couples (Sánchez Gassen and Perelli-Harris 2015). However, in Australia, unmarried couples have the same post-separation rights to property as divorced couples (Chigavazira et al. 2019).

At the same time, there are considerable differences across countries in other dimensions. For example, the UK and Australia are liberal welfare states (Esping-Andersen 1990) with less expansive welfare provision, means-tested benefits, and an important role assigned to the market. Social assistance is reserved for those in need. Financial support for families is available at a relatively low level and is typically targeted at reducing poverty, and childcare is largely provided by the private sector. Nonetheless, in the UK, extensive welfare support and social housing is available for single parents in need. It has been argued that Australia lies in between the liberal and the social-democratic welfare regime because it provides more extensive social support than liberal welfare regimes (Arts and Gelissen 2002). However, when it comes to family policies, it has been compared to the USA where no adequate parental leave policies or publicly funded childcare is available (Castles 2004). Germany and Belgium are conservative welfare states (Esping-Andersen 1990) where less emphasis is placed on the role of the market than in liberal welfare regimes (Thomas and Mulder 2016; Uunk 2004). Additionally, the level of financial support available for families depends on the employment status of the parents, the male breadwinner model is preferred, and marriage is encouraged through, for example, tax allowances. The Netherlands has many characteristics of the conservative as well as the social-democratic welfare regimes (Thomas and Mulder 2016). The latter is characterised by universal welfare provisions, a focus on socio-economic equality, and support for full employment (Uunk 2004).

### Gender Differences in Separation and Elevated Residential Mobility

Prior research on post-separation spatial mobility and housing outcomes suggests that separation is likely to have a different effect for men’s and women’s housing outcomes (Feijten 2005). Previous research has shown that women suffer more from separation or divorce financially than men (e.g. Aassve et al. 2007; Andreß et al. 2006; Jarvis and Jenkins 1999; Poortman 2000). This is because traditionally, there is a power imbalance within couples (Feijten and Mulder 2010), and women tend to be more engaged in unpaid domestic work, whereas men are more engaged in paid employment (Lersch and Vidal 2014). Thus, women have a weaker attachment to the labour market (no job, part-time job, or low paid jobs) (Feijten and Mulder 2010) and, hence, are less economically independent compared to men (Dewilde 2008; Feijten 2005). When a couple splits up, these gender imbalances and within-couple inequalities persist into the post-separation context (Feijten and Mulder 2010). Indeed, separated women are far more likely to bear primary responsibility for the caregiving of joint children and thus to cover the costs associated with childrearing, which are only offset to a small extent by any alimony paid by fathers (Dewilde 2008).

Previous research found that women are more likely to leave the joint home at separation than men although this seems to reverse when children are present (Ferrari et al. 2019; Fiori 2019; Mulder and Malmberg 2011; Mulder and Wagner 2010; Stone et al. 2014). Additionally, women were found to be more likely to move from owner-occupied to rental dwellings than men in the Netherlands (Feijten 2005), a finding attributed to the generally lower socio-economic independence of women. A recent study has shown for England and Wales that separated men and women primarily move to privately rented dwellings but that men also move to homeownership, whereas women are also likely to move to socially rented dwellings (Mikolai and Kulu 2018b).

The magnitude of elevated residential mobility among separated men and women is likely to depend on the institutional context. In countries, where extensive support is available for lone parents and public childcare is widely available, the income consequences of separation will be smaller for women than in countries where support to off-set women’s disadvantaged economic situation is not available (Dewilde 2008). Additionally, in countries where policies encourage the traditional male breadwinner model, women will be more dependent on men and we would expect to find more gendered post-separation residential mobility patterns than in countries where women are more attached to the labour force and are, hence, more economically independent.

### Hypotheses

Bringing the above arguments together, our hypotheses on the relationship between separation and residential mobility, and the moderating role of housing-market conditions, are as follows:

#### **H1**

*Elevated residential mobility*: Regardless of the housing market studied, separated individuals will experience higher levels of residential mobility than those who are in a marital or cohabiting relationship.

#### **H2**

*Trend towards stability*: Elevated mobility levels will decline with time since separation as people match their post-separation housing needs and consumption.

#### **H3**

*Cross-country variation*: The moderating role of housing-market conditions will lead to different durations of elevated post-separation residential mobility, with i) those living in Belgium experiencing the longest periods of post-separation instability, ii) those living in the Netherlands experiencing the shortest periods, and iii) those living in the UK, Germany, and Australia occupying intermediate positions.

#### **H4**

*Gender differences*: Overall, we expect to find gendered post-separation residential mobility patterns, although it is difficult to predict the magnitude of gender differences and how these vary across countries.

## Data

To investigate the impact of separation on residential mobility in a cross-national context, we use data from the following sources: the Household, Income, and Labour Dynamics in Australia (HILDA), the 2001 Belgian Census linked with the Population Register for the period 2001–2006, the German Socio-Economic Panel (SOEP), the Netherlands Kinship Panel Study (NKPS), and the British Household Panel Survey (BHPS) (Institute for Social and Economic Research 2010). Of these surveys, the HILDA, the SOEP, and the BHPS are highly comparable and follow similar data collection strategies. Residential histories were created using information on the year and month of moving to the current residence, as reported by the respondents. For the NKPS, the precise date of residential moves is not known: we only know whether respondents have changed residence since the previous wave. In the aforementioned datasets, the year and month^2^ of separation (i.e. the end of the relationship) were reported by the respondents. For Belgium, a 4% random sample was drawn of men and women who started a partnership (cohabitation or marriage) in 2000 or 2001. In the Belgian data, the identification of co-residential couples is based on the relationship between the head of the household and the other household members. The time of union dissolution is calculated by comparing the dates (day, month, and year) and destinations of residential moves of both partners between 2001 and 2006.^3^ All panel studies record one move per year. This implies that the rate of residential moves is likely to be slightly underestimated especially in the period immediately after separation, when individuals might move several times before finding suitable accommodation. The Belgian data allow for multiple moves within a calendar year. Additionally, all panel datasets contain retrospective union histories. It should be noted that the length of the observation window varies across countries, with information available for the following years: 2001–2013 for Australia; 2001–2005 for Belgium; 1990–2013 for Germany^4^; 1991–2013 for the Netherlands; and 1991–2008 for Britain. The analysis only includes unions formed after the start of the observation period.^5^

**Fig. 1:**
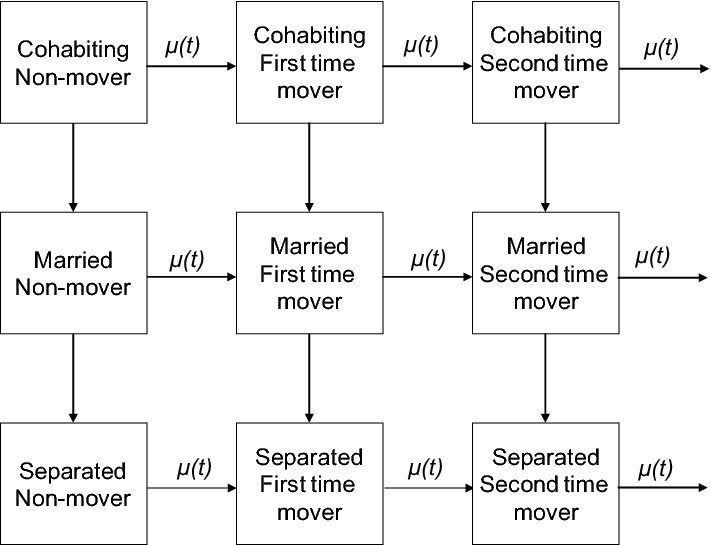
Processes and transitions. *Note μ*(*t*)—hazard or rate of residential change

At the start of the observation, individuals are in a relationship (cohabitation or marriage) and have not yet experienced a move during the observation window (Fig. 1). Partnership status is measured as a time-varying covariate. Individuals can change partnership status (from cohabiting to married or separated and from married to separated) and move simultaneously (several times). Individuals who form a new partnership following separation are included in the married or cohabiting category after the start of the new relationship. The risk population consists of individuals between the ages of 20 and 49. Individuals are censored at the last interview, at the death of the partner or at age 50, whichever comes first. Age 50 is chosen as the upper age limit because we would like to focus on couples whose children are still likely to be at home. Indeed, due to postponement of childbearing, some couples in their 50s still have under-aged children in the household. However, mobility rates significantly decrease over age (Rogers et al. 1983) and relatively few individuals change residence in their (early) 50s. Our further analysis on data from the UK showed that the inclusion of individuals over age 50 would not change the observed patterns. Mobility in and after the 60s is driven by different factors than in early- and mid-life (Falkingham et al. 2016). We use Poisson regression on aggregated occurrence–exposure data to analyse the rate of residential change by partnership status across countries with and without controlling for basic socio-demographic covariates (age, gender, calendar year, number of children, and educational level) (see below). The occurrence–exposure data are created by aggregating events (residential changes) and exposures (risk time) by the combination of covariates. If two or more events occur in the same month, the order of events is assumed to be as follows: separation, residential change, union formation. Such a sequence is needed to ensure natural order in partnership histories and to treat residential moves that happen simultaneously with changes in partnerships properly.

## Methods

For a comparative study of *n* countries regarding residential changes by partnership status, an option is to pool individual-level data from the countries and then fit a hazard regression model (Hoem et al. 2010). However, this is often not possible due to issues of data confidentiality: individual-level data cannot be released to another country or research group to conduct comparative analysis. For example, the Belgian population register data cannot be taken out of Belgium. It is possible to overcome this obstacle by using the count-data approach to compare residential mobility and migration rates across countries and population subgroups. Researchers need to prepare an event-time (or occurrence–exposure) table for each country, which is defined by a cross-classification over a set of time intervals and covariate categories (Hoem 1987; Preston 2005). The data for each cell in such a table include the total number of events, *E*_*jk*_; the total time (e.g. person-years or person-months) at risk, *R*_*jk*_; and values of covariates, *x*_*jk*_, for time period *j* and category *k*. For each cell, the ratio of the number of events to the risk time is a crude hazard or rate:

1 $$ \lambda_{jk} = {{E_{jk} } \mathord{\left/ {\vphantom {{E_{jk} } {R_{jk} }}} \right. \kern-\nulldelimiterspace} {R_{jk} }} $$

where *λ*_*jk*_ is the hazard for category *k* in time period *j*. Let *E*_*jk*_ denote the number of residential changes for group *k* in age group *j*. We treat *E*_*jk*_ as the realisation of a Poisson random variable with the mean *μ*_*jk*_:

2 $$ \mu_{jk} = \lambda_{jk} \times R_{jk} $$

The expected number of residential changes is, thus, the product of the hazard of residential change and exposure time. We can present this model in a log-linear format:

3 $$ \ln \;\mu_{jk} = \ln \;\lambda_{jk} + \ln \;R_{jk} $$

We then rearrange the equation to investigate the hazard of residential change:

4 $$ \ln \;(\mu_{jk} /R_{jk} ) = \ln \;\lambda_{jk} $$

Finally, we present a log-linear model for the hazard of residential changes, which also includes (additional) covariates:

5 $$ \ln \;\lambda_{jk} = \alpha_{j} + X_{k}^{\prime } \beta $$

where *α*_*j*_ = ln*λ*_*j*_ measures the hazard of residential changes by age (the ‘baseline’), **x′**_*k*_ is a vector of the covariates (e.g. partnership status, educational level, and calendar period), and ***β*** represents a vector of the parameters to measure their effects.

## Modelling Strategy

We estimate two sets of models. First, we focus on the relationship between partnership status and moves (Model 1). Second, in order to distinguish moves due to separation (i.e. event moves) from moves of separated individuals (i.e. state moves), and thus the potential period over which elevated residential mobility could persist, we split the category of separated individuals by time since separation (0–4 months after separation, 5–11 months after separation, and 12 or more months after separation^6^) (Model 2). This distinction is necessary because upon separation, at least one ex-partner has to move out of the joint home in order to separate. Thus, unless we made this distinction, mobility rates might be elevated not because separation leads to elevated moving rates but simply because by definition partners need to move apart in order to separate. The cut-off point between these categories is based on the distribution of moving risks over time since separation: the risk of a move remains high until four months after separation and somewhat decreases thereafter. We also experimented with other specifications, but the results remained robust.

Table 1 provides information on the number of events and risk time by partnership status in the five countries. For the Netherlands, only two time categories are available (0–11 months and 12+ months since separation) because the month of residential change was not recorded. To ensure that this specification does not bias the results, we conducted sensitivity analyses using only these two ‘time since separation’ categories for all countries (see Model 2b in Table 4 in “Appendix”). The samples include men and women from the same household. This implies that their partnership and residential histories are not independent. Therefore, we estimate all models separately for men and women. This strategy also enables us to identify potential gender differences in patterns of residential mobility of separated and partnered individuals across countries.

**Table 1:** Number of events and number and proportion of person-years by country and partnership status

	Number of events	Number (%) of person-years	Annual rate
Australia			
Cohabiting	3725	14,797 (50)	0.252
Married	1859	10,121 (34)	0.184
Separated	1711	4660 (16)	0.367
Separated 0–4 months	276	523 (11)^a^	0.528
Separated 5–11 months	440	932 (20)^a^	0.472
Separated 12 + months	995	3205 (69)^a^	0.310
Total	7295	29,577 (100)	0.247
Belgium			
Cohabiting	2564	21,738 (49)	0.118
Married	2126	17,569 (40)	0.121
Separated	1453	4896 (11)	0.297
Separated 0–4 months	316	878 (18)^a^	0.360
Separated 5–11 months	329	1097 (22)^a^	0.300
Separated 12+ months	808	2922 (60)^a^	0.277
Total	6143	44,204 (100)	0.139
Germany			
Cohabiting	4318	27,800 (42)	0.155
Married	3203	30,935 (46)	0.104
Separated	1663	8191 (12)	0.203
Separated 0–4 months	293	837 (10)^a^	0.350
Separated 5–11 months	302	1414 (17)^a^	0.214
Separated 12+ months	1068	5941 (73)^a^	0.180
Total	9184	66,925 (100)	0.137
The Netherlands			
Cohabiting	2063	11,894 (32)	0.173
Married	1745	20,840 (56)	0.084
Separated	633	4159 (11)	0.152
Separated 0–11 months	302	949 (23)^a^	0.318
Separated 12 + months	331	3210 (77)^a^	0.103
Total	4441	36,893 (100)	0.120
UK			
Cohabiting	1408	8994 (30)	0.157
Married	1496	15,706 (53)	0.095
Separated	1204	5025 (17)	0.240
Separated 0–4 months	227	425 (8)^a^	0.535
Separated 5–11 months	183	576 (11)^a^	0.318
Separated 12+ months	794	4,024 (80)^a^	0.197
Total	4108	29,725 (100)	0.138

^a^These proportions are calculated as the proportion of person-years among separated individuals. In Belgium, the cohabiting and married category is calculated using time-constant information on individuals’ partnership status between 2001 and 2006; the separated category is defined using time-varying information on whether couples still live in the same dwelling

*Source*: Australia: HILDA (2001–2013); Belgium: 2001 Belgian Census linked with the Population Register for 2001–2006; Germany: SOEP (1990–2013); the Netherlands: NKPS (1991–2013); UK: BHPS (1991–2008)

Married men and women in the UK are chosen as the reference category, and moving risks in all other groups and countries are compared to the levels of these groups. The main independent variable of interest is partnership status (cohabiting, married, and separated). Beyond this, we include controls for age [20–24 (reference), 25–29, 30–34, 35–39, 40–44, 45–49], order of partnership status [first (reference), second and higher order], residential status [non-mover (reference), moved once or more], and number of children [no children (reference), one child, two or more children]. We also control for calendar year [1990–1994 (reference), 1995–1999, 2000–2004, 2005–2009, 2010+] to account for changes in levels of residential mobility as well as changes in housing markets. Last, we control for educational level [low (reference), medium, high] to capture and adjust for socio-economic and compositional differences. As mentioned above, the length of observation window varies across countries. Still, there is a sufficient overlap in time periods across countries to make meaningful comparisons. We conducted sensitivity analyses using data from only the 2000s; as one would expect, the confidence intervals became wider, but the substantive results remained consistent.

## Results

### Descriptive Analysis: Mobility Rates by Age

**Fig. 2:**
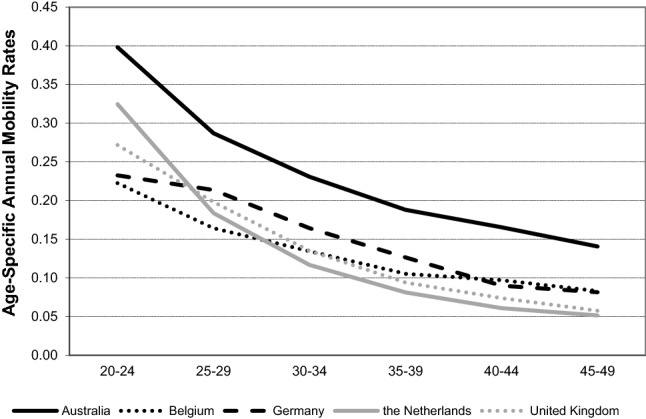
Annual mobility rates by age group and country. *Source*: Australia: HILDA (2001–2013); Belgium: 2001 Belgian Census linked with the Population Register for 2001–2006; Germany: SOEP (1990–2013); the Netherlands: NKPS (1991–2013); UK: BHPS (1991–2008)

We use survey data from four countries and linked census and register data from one country. To evaluate how comparable these five datasets are, Fig. 2 presents annual mobility rates for partnered (i.e. cohabiting and married) and separated individuals by age in five countries. (Further details are shown in Table 3 in “Appendix”.) Throughout the paper, we use the term ‘partnered individuals’ to jointly refer to married and cohabiting individuals. Note that our definition of mobility is a change of residence regardless of distance or geographical boundary. As expected, mobility rates decline by age across all countries. Mobility rates are relatively similar in the four European countries, whereas in Australia, they are approximately twice as high as in European countries. This is consistent with what previous studies have reported (Bell et al. 2015; Long 1991). Overall, across the four European countries, the mobility rates for partnered and separated individuals in ages 20 to 49 vary between 120 and 139 per 1000 person-years, while for Australia, this rate is 247 per 1000 person-years (see Table 3 in “Appendix”).

### Multivariate Analysis: Separation and Residential Mobility

**Fig. 3:**
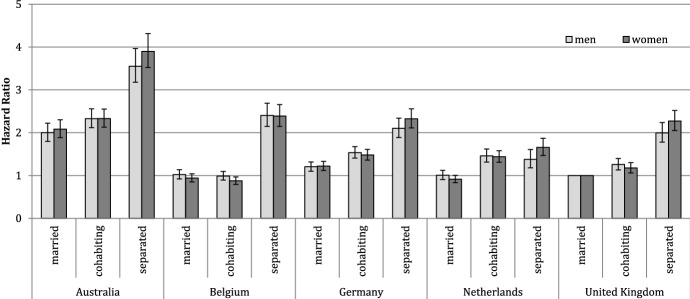
Risk of a residential move by country, gender, and partnership status (Model 1), hazard ratios. *Notes* The analysis controls for age, calendar period, number of children, educational level, number of moves, and number of unions. Whiskers indicate 95% confidence intervals compared with the reference category (married men and married women in the UK). In Belgium, the cohabiting and married category is calculated using time-constant information on individuals’ partnership status between 2001 and 2006; the separated category is defined using time-varying information on whether couples still live in the same dwelling. Source: Australia: HILDA (2001–2013); Belgium: 2001 Belgian Census linked with the Population Register for 2001–2006; Germany: SOEP (1990–2013); the Netherlands: NKPS (1991–2013); UK: BHPS (1991–2008)

Figure 3 shows the risk of a residential move by partnership status across countries for men and women. The figure presents hazards, relative to married individuals in the UK. We have fitted models separately for men and women and adjusted the rates for a set of covariates: age, calendar year, educational level, number of children, order of partnership, and order of residential move. As expected, mobility rates for married individuals are similar across European countries but twice as high in Australia. Cohabitants show somewhat higher moving risks than married individuals, except in Belgium.^7^ As expected, separated individuals have the highest mobility rates; they are about twice as likely to move as their married counterparts in all study countries.^8^ Interestingly, the patterns are very similar for men and women.

**Fig. 4:**
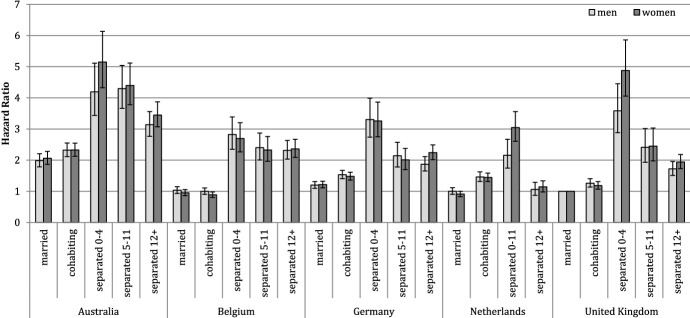
Risk of a residential move by country, gender, and partnership status; distinguishing separated category by time since separation (Model 2), hazard ratios. *Notes* The analysis is controlled for age, calendar period, number of children, educational level, number of moves, and number of unions. Whiskers indicate 95% confidence intervals compared with the reference category (married men and married women in the UK). In Belgium, the cohabiting and married category is calculated using time-constant information on individuals’ partnership status between 2001 and 2006; the separated category is defined using time-varying information on whether couples still live in the same dwelling. Source: Australia: HILDA (2001–2013); Belgium: 2001 Belgian Census linked with the Population Register for 2001–2006; Germany: SOEP (1990–2013); the Netherlands: NKPS (1991–2013); UK: BHPS (1991–2008)

Figure 4 shows the results of Model 2, where we distinguish between moves due to separation and moves of separated individuals by replacing the category of separated individuals with a variable showing time since separation. This model allows us to observe the change in mobility rates over time since separation and thus identify whether cross-country variation exists in post-separation residential instability. Consistent with our expectations, across all countries, the risk of a residential move is the highest shortly after separation and decreases gradually with time since separation. Individuals who separated recently in the UK and Australia are approximately four times as likely to move as married individuals in the UK, whereas in the remaining countries, they are approximately three times as likely to move.^9^ Interesting variations emerge in moving risks at longer durations since separation. Except for the Netherlands, moving risks remain relatively high even 12 or more months after separation. In Australia, Germany, and the UK, moving risks at this point remain twice those of married individuals. This suggests that separated individuals in these countries experience a degree of prolonged residential instability. Yet, instability seems particularly persistent in Belgium, where the risk of a move decreases only marginally with time. Belgium represents a country among the study countries where the opportunity to match housing consumption with needs seems particularly problematic. This is in contrast to the Netherlands where the housing market appears to offer the best conditions for recovering post-separation residential stability. Again, the patterns in this model are similar for men and women.

The effects of the control variables are as expected (see Table 2). Mobility rates are the highest in the early twenties, and they decline with age (Rogers et al. 1983). Mobility levels were elevated in the late 1990s and are slightly reduced thereafter, a result also consistent with previous studies (Champion and Shuttleworth 2017). Highly educated individuals are more likely to move than those with medium or low education, a common finding (e.g. Courgeau 1985; Mulder and Hooimeijer 1999) confirming human capital theories of migration (Sjaastad 1962). Men and women who have two or more children are less likely to move than those who do not have children. And finally, moving risks are higher among men and women who are in their second or subsequent unions and are lower among individuals who have already moved once (see Kulu 2008). These are all average effects, and they may vary across countries; however, additional analyses (not shown) have revealed that the effect of these control variables is remarkably similar across the studied countries.

**Table 2:** Relative risk of a residential move, women, and men

	Women		Men	
	Model 1	Model 2	Model 1	Model 2
Age				
20–24	1	1	1	1
25–29	0.725***	0.734***	0.785***	0.792***
30–34	0.567***	0.577***	0.618***	0.628***
35–39	0.428***	0.437***	0.504***	0.513***
40–44	0.341***	0.349***	0.387***	0.396***
45+	0.311***	0.319***	0.321***	0.328***
Period				
1990–1994	1	1	1	1
1995–1999	1.013	1.018	1.055	1.065*
2000–2004	0.899**	0.910*	0.956	0.969
2005–2009	0.848***	0.864***	0.901**	0.917*
2010+	0.747***	0.763***	0.787***	0.805***
Country * partnership status interactions				
Australia * cohabiting	2.331***	2.325***	2.327***	2.321***
Australia * married	2.083***	2.064***	2.000***	1.986***
Australia * separated	3.899***		3.550***	
Australia * separated 0–4 months		5.149***		4.192***
Australia * separated 5–11 months		4.398***		4.297***
Australia * separated 12+ months		3.446***		3.139***
Belgium * cohabiting	0.875**	0.887*	0.989	1.002
Belgium * married	0.940	0.953	1.021	1.034
Belgium * separated	2.388***		2.404***	
Belgium * separated 0–4 months		2.692***		2.821***
Belgium * separated 5–11 months		2.328***		2.400***
Belgium * separated 12+ months		2.363***		2.315***
Germany * cohabiting	1.481***	1.482***	1.535***	1.533***
Germany * married	1.219***	1.215***	1.205***	1.201***
Germany * separated	2.326***		2.102***	
Germany * separated 0–4 months		3.258***		3.304***
Germany * separated 5–11 months		2.009***		2.142***
Germany * separated 12+ months		2.241***		1.869***
Netherlands * cohabiting	1.439***	1.445***	1.460***	1.462***
Netherlands * married	0.915	0.911	1.008	1.005
Netherlands * separated	1.656***		1.379***	
Netherlands * separated 0–11 months		3.046***		2.157***
Netherlands * separated 12+ months		1.145		1.060
UK * cohabiting	1.177***	1.185**	1.257***	1.263***
UK * married	1	1	1	1
UK * separated	2.273***		1.996***	
UK * separated 0–4 months		4.875***		3.583***
UK * separated 5–11 months		2.449***		2.415***
UK * separated 12+ months		1.942***		1.719***
Number of children				
No children	1	1	1	1
One child	0.987	0.989	1.003	1.001
Two or more children	0.908***	0.906***	0.896***	0.892***
Educational level				
Low	1	1	1	1
Medium	0.984	0.983	0.947**	0.946*
High	1.107***	1.105***	1.066**	1.062*
Order of move				
No move	1	1	1	1
One or more moves	0.840***	0.847***	0.864***	0.870***
Order of union				
First union	1	1	1	1
Second- or higher-order union	1.456***	1.437***	1.398***	1.384***
Constant	0.018***	0.01***6	0.017***	0.015
Number of observations	10,534	16,837	10,251	16,459***
Log-likelihood	-10,660	-12,159	-9491	-10,706
LR χ^2^	5386	5599	3760	3873
Prob > χ^2^	0.000	0.000	0.000	0.000

The time unit is person-month. *p < 0.05; **p < 0.01; ***p < 0.001. In Belgium, the cohabiting and married category is calculated using time-constant information on individuals’ partnership status between 2001 and 2006; the separated category is defined using time-varying information on whether couples still live in the same dwelling

*Source*: Australia: HILDA (2001–2013); Belgium: 2001 Belgian Census linked with the Population Register for 2001–2006; Germany: SOEP (1990–2013); the Netherlands: NKPS (1991–2013); UK: BHPS (1991–2008)

We also estimated a set of interaction models (results not shown but available upon request) to explore educational differences between individuals’ post-separation residential mobility patterns and to investigate whether the patterns might be different for childless individuals and those who have children. We found largely similar patterns by level of education and parenthood status.

## Conclusions

This paper investigated the relationship between separation and elevated residential mobility in five high-income countries: Australia, Belgium, Germany, the Netherlands, and the UK. We distinguished between moves due to separation and moves of separated individuals to determine the degree to which separated individuals experience prolonged elevated residential mobility (characterised by the undertaking of repeated ‘adjustment’ moves). As expected, our analysis showed that separated individuals are considerably more likely to move than partnered individuals. Mobility levels were the highest shortly after separation and decreased with time since separation; however, they remained relatively high even a year after separation. These findings are in line with our expectations (H1 and H2) and indicate that separation leads to prolonged residential instability in the study countries.

We also showed that the extent to which mobility levels decline with time since separation varies across countries. In Australia, Germany, and the UK, we observed a gradual decline in post-separation mobility rates, although even a year after separation, the risk of a move among separated individuals remained higher than among partnered individuals. However, in Belgium, the differences between separated and partnered individuals were particularly large and persistent, with little decline in post-separation mobility rates over time. In contrast, the differences between separated and partnered individuals were the smallest in the Netherlands and declined rapidly with time since separation; a year after separation mobility levels of separated individuals were almost the same as for partnered individuals. These findings seem to support that wider structural constraints and opportunities play an important role in the residential (in)stability of separated individuals. While separation leads to elevated mobility levels by definition in the short term (i.e. at least one of the partners have to move out of the joint home), the patterns we observe suggest that context plays a crucial role in moderating the duration over which subsequent residential (in)stability is experienced. In line with our expectation (H3), we find that housing markets where access to homeownership is difficult and the availability of social housing is limited (i.e. Belgium) are associated with the longest periods of post-separation instability, whereas housing markets offering affordable and good-quality rental accommodation (i.e. the Netherlands) are associated with the shortest periods of residential instability.

Our analysis thus highlights the importance of housing markets in shaping residential mobility among separated individuals. At the one end of the spectrum are countries where access to mortgages is constrained to the wealthiest segment of society and where costs of purchasing a home are normally financed from savings, family help, or inheritance; the rental sector is often small and a significant deposit or advance payments are required. At the other end of the spectrum are countries where access to mortgages is relatively easy and the rental sector is well developed including opportunities for social renting for those in need. Clearly, separated individuals have much wider opportunities in the latter group of countries; some may be able to purchase a new (small) home; others (the majority) will be able to quickly find opportunities in the private or social rental sector. In contrast, in countries with limited housing opportunities, separated individuals may need to live in temporary accommodation (e.g. relatives) and are likely to experience long-term residential instability.

However, there are several arguments, which may challenge our interpretation of the results, especially elevated post-separation residential mobility in Belgium. First, it is possible that moves out of the joint home are delayed because it takes time to sell the joint property and this process may vary across countries. To address this issue, we investigated separately first- and second-order or higher-order moves of separated individuals (results available upon request). Although the decline in the risk of a first move over time since separation seemed to be slightly slower in Belgium than in the other countries, the levels of second- or higher-order moves remained elevated in Belgium even a year after separation suggesting that ‘adjustment moves’ rather than delayed moves out of the joint home are the main reason behind (prolonged) elevated post-separation residential mobility in Belgium (although it is still possible that a slightly different measurement of moves may explain some elevated mobility among the separated in Belgium, i.e. multiple moves within a year were allowed in the Belgian, but not in other data sets). Another possible explanation for the differences in residential mobility of separated individuals across countries could be the different speed and levels of repartnering. We explored this possibility further, but our analysis (not shown) did not support this argument. Third, our further analysis indicated that (prolonged) elevated post-separation residential mobility in Belgium may be more common among low-educated individuals. Although highly educated separated individuals (also) exhibited elevated mobility levels a year after separation, elevated mobility rates were more pronounced among those with the lowest levels of education. We also investigated educational differences in post-separation residential mobility patterns of men and women but did not detect significant differences between low/medium- and highly educated individuals. Similarly, we did not find differences in the patterns by whether individuals were childless or had at least one child.

Surprisingly, we did not find many differences in post-separation residential mobility by gender. The reason for the lack of differences might be related to small sample size, especially when time since separation was also considered. Women are often in a weaker economic position than men, but children normally stay with mothers after divorce. Previous research suggests that women have a higher propensity to leave the joint home upon separation than men, but when children are present, men have higher propensities to move out of the joint home (Ferrari et al. 2019; Fiori 2019; Mulder and Malmberg 2011; Mulder and Wagner 2010; Stone et al. 2014). Further, gender differences may become visible only when we study the housing tenure and type individuals move to following separation. Previous research shows that men are more likely to remain or become homeowners after separation and to move to smaller dwellings, whereas many women stay in or move to social renting and are likely to move to larger dwellings (Mikolai and Kulu 2018a, b, 2019).

Overall, the findings indicate that moving following separation is a universal phenomenon among all socio-economic groups as well as among parents and childless ex-couples. These factors might be more important for individuals’ post-separation housing tenure. For example, Mikolai and Kulu (2019) showed for Britain that highly educated separated individuals are likely to remain homeowners following separation, whereas those with low levels of education tend to stay in or move to socially rented dwellings. Similarly, Mikolai and Kulu (2018b) found that low-educated separated women were most likely to move to social renting, whereas those with high levels of education were the least likely to do so. Furthermore, in a comparative study, Mikolai et al. (2019) showed that educational level is important in predicting the housing tenure of both partnered and separated individuals; highly educated people tend to move to homeownership, whereas those with lower levels of education move to social renting regardless of partnership status.

Regarding the role of parenthood, those who have at least one child are likely to remain in social renting after separation in Britain (Mikolai and Kulu 2019). The role of children seems especially important for separated women; those who have children tend to move to socially or privately rented dwellings, whereas childless women are most likely to move to private renting followed by moving in with family or friends (Mikolai and Kulu 2018b). Parenthood status has also been shown to be an important factor for the residential experiences of separated individuals in other industrialised countries; separated parents had somewhat higher moving risks to social and private renting than childless individuals (Mikolai et al. 2019).

We have proposed a novel way of distinguishing between moves due to separation and moves of separated people which enabled us to measure the persistence of elevated post-separation residential mobility in different national contexts. A further methodological novelty relates to the use of Poisson regression models on aggregated occurrence–exposure data. This approach enables us to calculate mobility rates for partnered and separated individuals in different countries adjusted for a set of covariates. Increasing availability of high-quality longitudinal data has significantly enhanced the opportunities for comparative research. However, the pooling of individual-level survey data from different countries has remained difficult due to confidentiality issues. This is especially the case when register data are used, which cannot be shared across research groups or released to researchers residing in another country. Thus, our approach represents a way of overcoming the common issues that confront many researchers involved in comparative work.

Recent studies have shown that individual-level unobserved characteristics jointly influence partnership transitions and residential mobility (e.g. Lersch and Vidal 2014; Mikolai and Kulu 2018a, b). In this study, we were not able to account for such unobserved characteristics because individual-level data could not be shared across the research teams. Nonetheless, estimating the risk of a move by partnership status across five countries using pooled analysis enables us to directly compare moving risks across countries which would not be possible using country-specific analyses. Future research should estimate simultaneous event history models separately for each country using individual-level data to find out about the extent to which such unobserved factors jointly influence partnership transitions and residential mobility in the study countries. Another option might be to use the approach offered by data shield (Wolfson et al. 2010).

To summarise, combining high-quality longitudinal data from five countries, this study showed that separated men and women are significantly more likely to move than cohabiting and married individuals. The risk of residential change is the highest shortly after separation, and it decreases with duration since separation. However, the magnitude of this decline varies by housing context suggesting that housing markets mediate the relationship between separation and spatial mobility. We have shown that in most study countries, separation leads to prolonged elevated residential mobility which could lead to instabilities in psychological well-being, and children’s schooling. Thus, a greater awareness of post-separation residential instability, its triggers, conditions, and outcomes, could be important for developing new insights into post-separation recovery processes and the formation of related policies.

## Appendix

See Tables 3 and 4.

**Table 3:** Number of events, person-years, and annual mobility rates by country and age

	Number of events	Number of person-years	Annual rate
Australia			
20–24	1858	4664	0.398
25–29	2009	7005	0.287
30–34	1471	6374	0.231
35–39	956	5086	0.188
40–44	624	3772	0.165
45–49	377	2682	0.141
Total	7295	29,582	0.247
Belgium			
20–24	917	4118	0.223
25–29	2174	13,237	0.164
30–34	1599	11,883	0.135
35–39	715	6792	0.105
40–44	415	4282	0.097
45–49	323	3892	0.083
Total	6143	44,204	0.139
Germany			
20–24	550	2366	0.232
25–29	2113	9890	0.214
30–34	2454	14,938	0.164
35–39	2009	15,897	0.126
40–44	1252	13,931	0.090
45–49	806	1572	0.513
Total	9184	58,594	0.157
The Netherlands			
20–24	841	2590	0.325
25–29	1313	7146	0.184
30–34	1054	9024	0.117
35–39	663	8171	0.081
40–44	373	6135	0.061
45–49	197	3828	0.051
Total	4441	36,893	0.120
UK			
20–24	965	3551	0.272
25–29	1224	6181	0.198
30–34	852	6309	0.135
35–39	531	5654	0.094
40–44	335	4537	0.074
45–49	201	3493	0.058
Total	4108	29,725	0.138

*Source*: Australia: HILDA (2001–2013); Belgium: 2001 Belgian Census linked with the Population Register for 2001–2006; Germany: SOEP (1990–2013); the Netherlands: NKPS (1991–2013); UK: BHPS (1991–2008)

**Table 4:** Results of the sensitivity analysis

	Women	Men
	Model 2b	Model 2b
Age		
20–24	1	1
25–29	0.734***	0.792***
30–34	0.577***	0.628***
35–39	0.437***	0.513***
40–44	0.349***	0.396***
45+	0.319***	0.328***
Period		
1990–1994	1	1
1995–1999	1.017	1.064*
2000–2004	0.909**	0.968
2005–2009	0.863***	0.915*
2010 +	0.762***	0.803***
Country * partnership status interactions		
Australia * cohabiting	2.326***	2.322***
Australia * married	2.065***	1.987***
Australia * separated 0–11 months	4.678***	4.261***
Australia * separated 12 + months	3.450***	3.142***
Belgium * cohabiting	0.886*	1.000
Belgium * married	0.952	1.033
Belgium * separated 0–11 months	2.490***	2.586***
Belgium * separated 12+ months	2.362***	2.313***
Germany * cohabiting	1.481***	1.533***
Germany * married	1.215***	1.201***
Germany * separated 0–11 months	2.482***	2.585***
Germany * separated 12+ months	2.242***	1.870***
Netherlands * cohabiting	1.446***	1.462***
Netherlands * married	0.912	1.005
Netherlands * separated 0–11 months	3.047***	2.158***
Netherlands * separated 12+ months	1.145	1.061
UK * cohabiting	1.185**	1.263***
UK * married	1	1
UK * separated 0–11 months	3.456***	2.901***
UK * separated 12+ months	1.943***	1.719***
Number of children		
No children	1	1
One child	0.988	1.001
Two or more children	0.906***	0.892***
Educational level		
Low	1	1
Medium	0.983	0.946**
High	1.105***	1.062**
Order of move		
No move	1	1
One or more moves	0.846***	0.869***
Order of union		
First union	1	1
Second- or higher-order union	1.439***	1.385***
Constant	0.016***	0.015***
Number of observations	13,813	13,435
Log–likelihood	– 11,646	– 10,228
LR χ^2^	5550	3852
Prob > χ^2^	0.000	0.000

The time unit is person-month. **p* < 0.05; ***p* < 0.01; ****p* < 0.001. In Belgium, the cohabiting and married category is calculated using time-constant information on individuals’ partnership status between 2001 and 2006; the separated category is defined using time-varying information on whether couples still live in the same dwelling

*Source*: Australia: HILDA (2001–2013); Belgium: 2001 Belgian Census linked with the Population Register for 2001–2006; Germany: SOEP (1990–2013); the Netherlands: NKPS (1991–2013); UK: BHPS (1991–2008)

## Footnotes

1 https://e-justice.europa.eu/home.do.

2 In wave 1 of the NKPS, only the year of separation was reported by the respondents.

3 The limitation of the Belgian data is that we know whether individuals were cohabiting or married in 2001 and 2006, but we do not know the date of marriage for cohabitants. Whereas this may influence the results related to mobility levels of cohabiting and married individuals, it is unlikely to bias the results related to mobility rates of separated individuals.

4 Although the SOEP started in 1984, in this study, we only use information from 1990 onwards to harmonise the start date of the observation period across different data sources.

5 The analyses exclude never partnered single individuals. The main reason for this is to simplify data preparation and increase data comparability across countries. Moreover, it has been shown elsewhere for the UK (Mikolai and Kulu 2018a, b) that separated men and women have higher moving risks than never partnered single men and women. In other words, separated individuals’ post-separation residential mobility patterns do not simply reflect processes that apply to single people in general.

6 We used 12+ months as a cut-off point because using a more detailed measure of time since separation, previous studies (Feijten and van Ham 2010; Mikolai and Kulu 2018a, b) have shown for the UK that levels of residential mobility do not change much after 12+ months.

7 This may be related to the construction of the partnership status variable in Belgium, which uses time-constant information on whether individuals were married or cohabiting between 2001 and 2006.

8 We have also estimated country-specific models (available upon request) to show that separated men and women are significantly more likely to experience a residential move in all countries than their married counterparts.

9 Again, we have estimated country-specific models (available upon request), which show that in each country, separated men and women have higher moving risks than their married counterparts and that this relationship holds also as time since separation increases. An exception are the moving risks of Dutch men who have separated 12 or more months ago; the moving risks of these men are not significantly different from that of their married counterparts.
